# Synthesis of cobalt nanowires in aqueous solution under an external magnetic field

**DOI:** 10.3762/bjnano.7.91

**Published:** 2016-07-07

**Authors:** Xiaoyu Li, Lijuan Sun, Hu Wang, Kenan Xie, Qin Long, Xuefei Lai, Li Liao

**Affiliations:** 1 School of Chemical Engineering, Sichuan University, Chengdu 610065, PR China

**Keywords:** aqueous solution, cobalt nanowires, external magnetic field, magnetic properties, surfactant

## Abstract

In contrast to the majority of related experiments, which are carried out in organic solvents at high temperatures and pressures, cobalt nanowires were synthesized by chemical reduction in aqueous solution with the assistance of polyvinylpyrrolidone (PVP) as surfactant under moderate conditions for the first time, while an external magnetic field of 40 mT was applied. Uniform linear cobalt nanowires with relatively smooth surfaces and firm structure were obtained and possessed an average diameter of about 100 nm with a coating layer of PVP. By comparison, the external magnetic field and PVP were proven to have a crucial influence on the morphology and the size of the synthesized cobalt nanowires. The prepared cobalt nanowires are crystalline and mainly consist of cobalt as well as a small amount of platinum. Magnetic measurements showed that the resultant cobalt nanowires were ferromagnetic at room temperature. The saturation magnetization (*M*_s_) and the coercivity (*H*_c_) were 112.00 emu/g and 352.87 Oe, respectively.

## Findings

In recent years, cobalt nanowires, as a ferromagnetic material, have attracted considerable attention due to their outstanding magnetic properties and excellent performance in applications in high-density magnetic storage media [1–2], in immune magnetic separation [3], in gene delivery [4] and as targeted drug carrier [5]. Hydrothermal and solvothermal methods are well-developed approaches to fabricate cobalt nanowires [6–10]. However, such methods set high requirements for the equipment because of high temperatures and pressures. Therefore, researches concerning a solution-reduction method under moderate conditions for preparing cobalt nanowires have been carried out due to their simplicity and low cost [11–12]. Organics, such as ethylene glycol and propylene glycol, are used to serve as solvents in most of the experiments. They can provide stable process for preparing nanowires owing to the relatively high viscosity of these solvents. Reactions proceed gently in organic solvents, which results in a high degree of crystallization and a high dispersibility of the nanowires. Nevertheless, organic solvents are not easily washed out of products, and the most important problem is that they are not environmentally friendly. Compared to organic solvents, the use of water as the solvent is cheaper and more convenient and does less harm to the environment. There are few reports on preparing cobalt nanowires in aqueous solution up to now [13]. In our previous studies, we have successfully prepared nickel and nickel/copper nanowires [14–16], and cobalt nanowires were synthesized using NaBH_4_ as initiator without surfactant in ethylene glycol solution with an external magnetic field under moderate conditions [12]. In this research, with polyvinylpyrrolidone (PVP) as surfactant, uniform linear cobalt nanowires with a mean diameter of about 100 nm were synthesized in aqueous solution under an external magnetic field at low temperature and atmosphere pressure for the first time.

All the chemical reagents used in the experiment were analytical grade. CoCl_2_·6H_2_O, EDTA-2Na and PVP acted as Co precursor, chelating agent and surfactant, respectively. N_2_H_4_·H_2_O and H_2_PtCl_6_·6H_2_O worked as reductant and initiator, respectively. The synthesis was performed inside water bath set at 80 °C. Firstly, 6 mmol CoCl_2_·6H_2_O and 6 mmol EDTA-2Na were dissolved in 60 mL distilled water inside a polytetrafluoroethylene beaker placed between two magnets, with an applied field of 40 mT measured by a HT20 tesla meter. Then NaOH was added to adjust the pH value of the solution to about 14, and 0.18 g PVP was dissolved in the solution by thorough stirring. Next, 0.30 mL N_2_H_4_·H_2_O and 0.12 mL of 0.0253 M H_2_PtCl_6_·6H_2_O were mixed with the solution. The reaction proceeded for 15 min, during which nanowires were formed gradually. Finally, the prepared nanowires were separated from the solution with a magnet, and washed ultrasonically in distilled water and ethanol three times each to remove the organics from the surface of the nanowires.

The size and morphology of the synthesized nanowires were observed with a field emission scanning electron microscope (SEM, JEOL JSM-7500F) and a transmission electron microscope (TEM, JEOL JEM-100CX). The composition and crystal structure of the freeze-dried products were characterized by energy dispersive spectrometry (EDS, Oxford Instruments X-Max 51-XMX0019), X-ray diffraction (XRD, Philips X'Pert Pro MPD) in the range from 20–90° using Cu radiation (λ = 0.154249 nm) with an incremental step size of 4°/min, the generator voltage of 40 kV and tube current of 40 mA, and selected area electron diffraction (SAED, FEI Tecnai-G20). The magnetic properties, such as saturation magnetization (*M*_s_) and coercivity (*H*_c_), were studied by a magnetic property measurement system superconducting quantum interference device (Quantum Design, Inc., MPMS SQUID XL) at room temperature using an applied field of up to 2.5 T.

**Figure 1:**
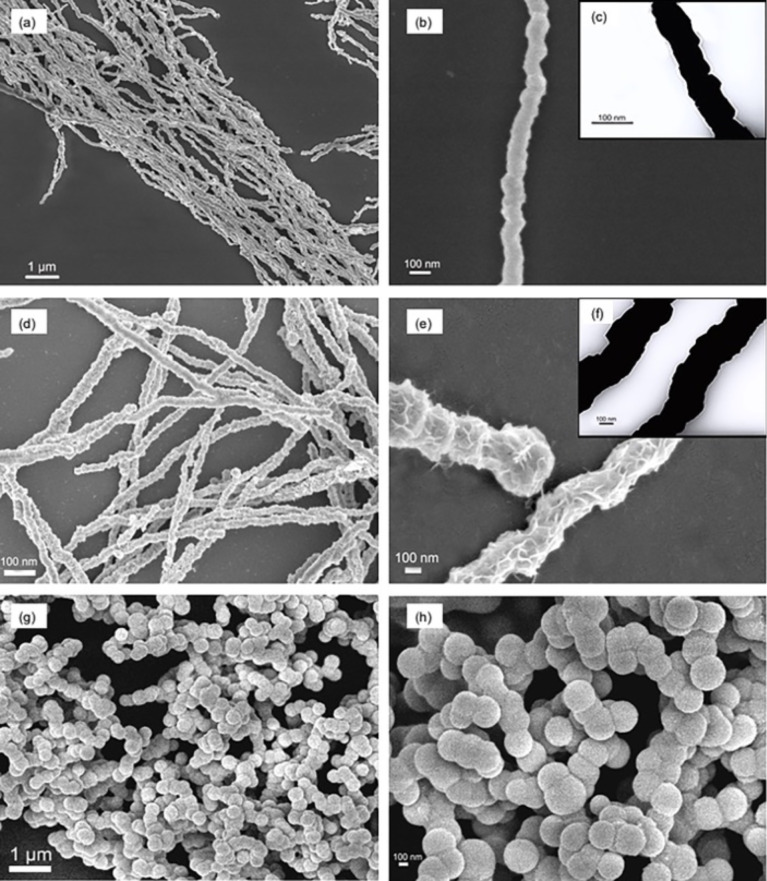
SEM images of cobalt nanowires prepared under an external magnetic field with PVP (a,b) and without PVP (d,e), and cobalt nanowires prepared without an external magnetic field (g,h). The insets (c, f) show the corresponding TEM images of cobalt nanowires (b,e), respectively.

Figure 1a,b show SEM images of cobalt nanowires prepared with PVP in aqueous solution under an external magnetic field. Uniform linear cobalt nanowires with a mean diameter of about 100 nm were observed, which were much smaller than those prepared in our previous studies in diameter (about 500 nm) [12]. The cobalt nanowires possessed relative smooth surface without apparent aggregation. Figure 1c shows the TEM image of the PVP-protected cobalt nanowires prepared under an external magnetic field. The nanowires exhibited a firm linear structure without any gap. Furthermore, the diameter of the nanowire shown in the TEM image is about 60 nm, while that in Figure 1b is about 100 nm. The obvious difference in diameter observed by SEM and TEM indicated that synthesized cobalt nanowires were uniformly coated by a layer of PVP whose thickness was about 20 nm. Figure 1d–f shows SEM and TEM images of cobalt nanowires prepared without PVP in aqueous solution under an external magnetic field. In the absence of PVP, nanowires with a relatively rough surface were obtained, whose size distribution was in the range of 200 to 350 nm. There were also some chain-like structures observed by SEM. It demonstrated that PVP, as a surfactant, had a crucial influence on the morphology and the size of cobalt nanowires synthesized via this method. Figure 1g,h shows SEM images of cobalt nanowires prepared without an external magnetic field. In the absence of an external magnetic field, only spherical particles with the diameter of about 250 nm were obtained, which aggregated without an obvious orientation. Comparing the diameter with that of the nanowires in Figure 1b, it can be inferred that applying an external magnetic field could inhibit the growth of the products. During the experimental process, small cobalt nanoparticles were generated in the solution at first, regardless of the presence of the external magnetic field. Then these small nanoparticles agglomerated and formed larger particles that started to align along the external magnetic field. As the reaction proceeded, Co(II) ions in the solution were reduced and deposited in the gaps between particles, resulting in the formation of cobalt nanowires [11,14].

**Figure 2:**
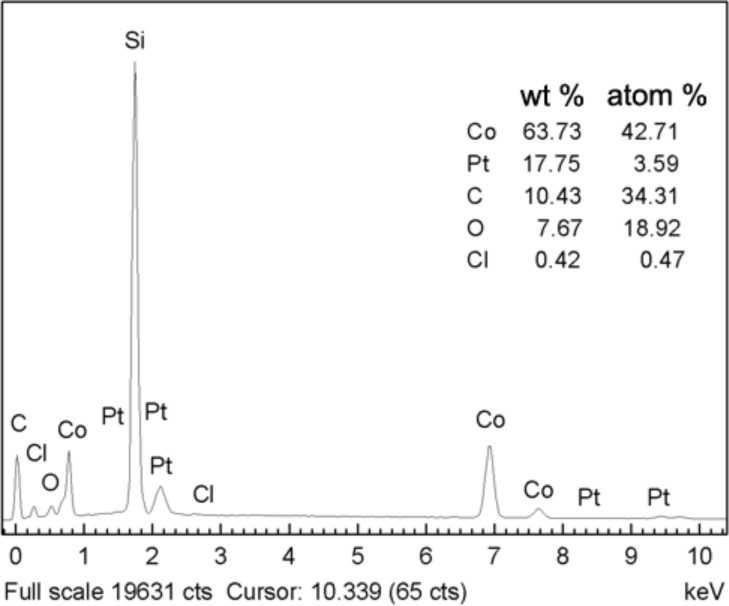
EDS spectrum of the PVP-protected cobalt nanowires prepared under an external magnetic field. Silicon was left out from the composition evaluation for the sake of clarity.

Figure 2 shows the EDS spectrum of the PVP-protected cobalt nanowires prepared under an external magnetic field. Weight percent (wt %) and atom percent (atom %) of each element were determined by EDS analysis. The silicon peak stems from the silicon wafer substrate. For the sake of clarity it was left out from the calculation of the elemental composition. Cobalt and platinum acting as heterogeneous nucleation sites are clearly seen. Carbon and oxygen can be ascribed to the PVP coating on the nanowires and, to some extent, to CO_2_ adsorbed on nanowires during the preparation of samples. There is also very little residual chlorine detected, which comes from H_2_PtCl_6_·6H_2_O.

**Figure 3:**
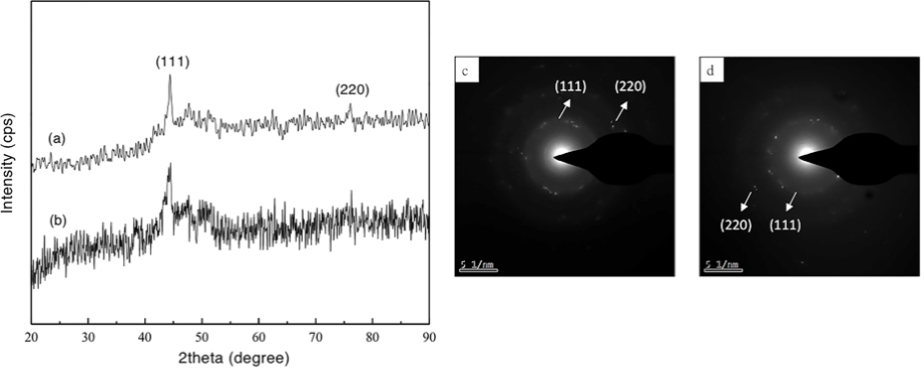
XRD patterns of cobalt nanowires prepared with PVP (a) and without PVP (b) under an external magnetic field. The corresponding SAED patterns of cobalt nanowires prepared with PVP (c) and without PVP (d).

XRD patterns of the cobalt nanowires prepared with PVP and without PVP under an external magnetic field are shown in Figure 3a,b, respectively. The corresponding SAED patterns are shown in Figure 3c,d. The diffraction peaks of cobalt nanowires prepared with PVP and without PVP could be indexed with the reflections of face-centered cubic (fcc) Co (PDF standard cards, JCPDS 15-0806, space group *Fm*−3*m*). Two peaks of fcc Co (2θ = 44.43° and 2θ = 75.94°) corresponding to Miller indices (111) and (220), respectively, were observed in each XRD pattern, and a diffraction ring as well as some scattered diffraction mottling were shown in each SAED pattern, which demonstrated that the resultant nanowires possessed crystal structure and PVP had only little impact on that.

**Figure 4:**
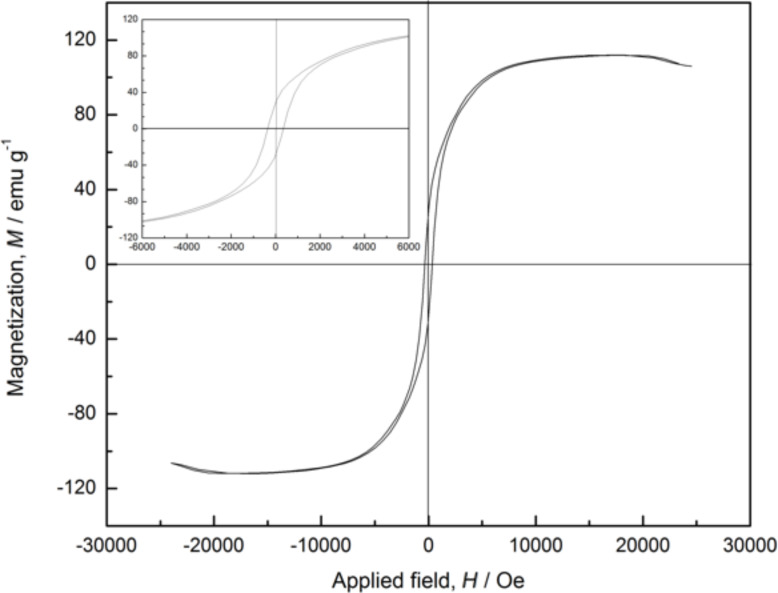
The hysteresis loop of the PVP-protected cobalt nanowires prepared under an external magnetic field measured at room temperature. The inset shows the respective expanded plots for fields between −6000 and 6000 Oe.

Figure 4 displays the hysteresis loop measured at room temperature under an applied magnetic field of up to 25000 Oe for the PVP-protected cobalt nanowires obtained in aqueous solution under an external magnetic field of 40 mT. An expanded plot is shown in the insert for field strengths between −6000 Oe and 6000 Oe. The hysteresis loop suggested that the synthesized cobalt nanowires were ferromagnetic at room temperature, which differs from the superparamagnetic property exhibited in the previous report [13]. The coercivity (*H*_c_) of the cobalt nanowires were 352.87 Oe, and the saturation magnetization (*M*_s_) were 112.00 emu g^−1^, which was lower than the corresponding value of bulk cobalt (162.5 emu g^−1^) [11].

In summary, uniform linear cobalt nanowires with a mean diameter of about 100 nm were obtained by chemical reduction in aqueous solution with an external magnetic field for the first time. The cobalt nanowires exhibited a relatively smooth surface and firm structure with a layer of PVP, which had a significant impact on the morphology and size of nanowires. The synthesized nanowires mainly consisted of cobalt and a small amount of platinum with crystal structure. Cobalt nanowires prepared were ferromagnetic at room temperature. The saturation magnetization (*M*_s_) and the coercivity (*H*_c_) were 112.00 emu/g and 352.87 Oe, respectively.
